# Cryo‐EM map interpretation and protein model‐building using iterative map segmentation

**DOI:** 10.1002/pro.3740

**Published:** 2019-10-24

**Authors:** Thomas C. Terwilliger, Paul D. Adams, Pavel V. Afonine, Oleg V. Sobolev

**Affiliations:** ^1^ Los Alamos National Laboratory Los Alamos New Mexico; ^2^ New Mexico Consortium Los Alamos New Mexico; ^3^ Molecular Biophysics & Integrated Bioimaging Division Lawrence Berkeley National Laboratory Berkeley California; ^4^ Department of Bioengineering University of California Berkeley Berkeley California

**Keywords:** cryo‐electron microscopy, map interpretation, map segmentation, model‐building

## Abstract

A procedure for building protein chains into maps produced by single‐particle electron cryo‐microscopy (cryo‐EM) is described. The procedure is similar to the way an experienced structural biologist might analyze a map, focusing first on secondary structure elements such as helices and sheets, then varying the contour level to identify connections between these elements. Since the high density in a map typically follows the main‐chain of the protein, the main‐chain connection between secondary structure elements can often be identified as the unbranched path between them with the highest minimum value along the path. This chain‐tracing procedure is then combined with finding side‐chain positions based on the presence of density extending away from the main path of the chain, allowing generation of a C_α_ model. The C_α_ model is converted to an all‐atom model and is refined against the map. We show that this procedure is as effective as other existing methods for interpretation of cryo‐EM maps and that it is considerably faster and produces models with fewer chain breaks than our previous methods that were based on approaches developed for crystallographic maps.

## 1 INTRODUCTION

Major technological improvements have been made in single‐particle electron cryo‐microscopy (cryo‐EM) over the past few years. In particular, the development of electron‐counting detection has allowed corrections for time‐dependent image shifts that previously limited the effective resolution of cryo‐EM and further improvements are likely.^1^, ^2^ It is now possible for cryo‐EM to be readily applied to macromolecular structure determination at resolutions of 4.5 Å or better.^3^, ^4^ At these resolutions, structural details such as the path of a protein backbone and shapes of side‐chains can be visible. A rapid increase in the rate of moderate‐to‐high resolution structure determination by cryo‐EM has resulted, as illustrated by deposits to the Protein Data Bank (PDB).^5^ For example, in 2014, there were 192 deposits of structures in the resolution range of 3.5–4.0 Å in the PDB, and 20 were from cryo‐EM. In 2018, there were 419 deposits of structures in this range in the PDB, and 287 of these were determined by cryo‐EM.

An important step in the interpretation of a cryo‐EM map is the construction of an atomic model representing the structure of the macromolecule that has been imaged. If an existing model is available (e.g., from a previous cryo‐EM or crystal structure) this interpretation is typically carried out by docking the existing model in the map and then adjusting the model to match as well as possible both the map and expected molecular geometry (e.g., 6). If no model is available, interpretation is considerably more difficult. In such a case, the map is usually interpreted by building a new (ab initio) model based on the features visible in the map and the sequence of the macromolecule.

In this work, we focus on maps with resolutions in the range of 4.5 Å or better. In this resolution range side chains are often visible and the main chain can be relatively well‐defined, so that automatic map interpretation tools can be effective. There are now a number of tools available for ab initio model‐building based on a cryo‐EM map in this resolution range. The visual modeling tool *Coot* 7 has substantial automation and allows a researcher to direct the building process. Automatic modeling tools have been developed that can generate models requiring very few or no choices to be made by the researcher.^8^ Some methods focus on secondary structure identification.^9^, ^10^, ^11^, ^12^ Other methods combine *Rosetta* structure‐modeling with cryo‐EM model‐building.^13^, ^14^, ^15^ Further semi‐automated tools exist for full map interpretation,^16^ and automated tools have been developed that use chain‐tracing^17^, ^18^ and template‐matching approaches,^19^, ^20^ including tools that were originally developed for crystallographic map interpretation.^21^ Although these tools are very powerful for cryo‐EM map interpretation, so far none exist that can reliably produce a complete model without sequence register errors and main‐chain insertions and deletions for structures with resolutions worse than about 3 Å. Consequently, there remains a need for new approaches to automatic map interpretation, and particularly for approaches that are suited to this challenging resolution range of 3–4.5 Å.

**Figure 1:**
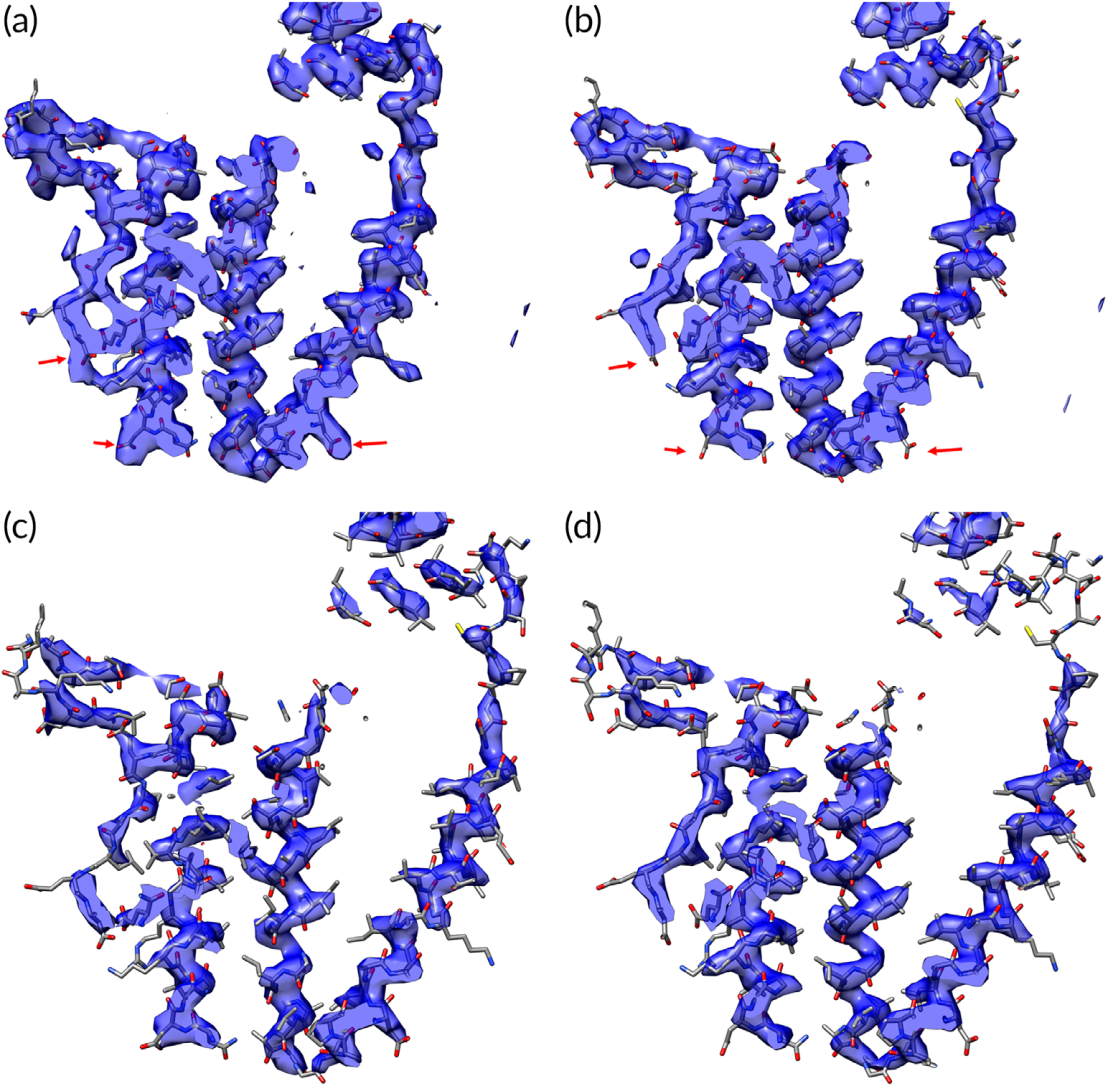
Experimental X‐ray and cryo‐EM maps of groEL. (a) Experimental X‐ray map (MIR phasing, density modified) at resolution of 3.8 Å of groEL showing residues 10–170 (masked around atoms in these residues). (b) Experimental cryo‐EM map at resolution of 3.5 Å of groEL showing residues 10–170 (masked around atoms in these residues). (c) X‐ray map of groEL from (a), but at higher contour level. (d) Cryo‐EM map of groEL from (b), but at higher contour level. The arrows indicate locations of aspartate and glutamate side chains where the density appears to be substantially different between the X‐ray and cryo‐EM maps

As many of the existing cryo‐EM model‐building approaches were originally developed for interpretation of maps from X‐ray analyses, it is worth considering whether methods for crystal structure interpretation should be expected to work equally well for cryo‐EM maps. The technologies for obtaining X‐ray and cryo‐EM maps are very different, one involving scattering of X‐rays and the other scattering of electrons. Consequently, some differences are expected due to the intrinsic differences in scattering, and additionally, differences could occur due to differences in radiation damage effects.^22^, ^23^, ^24^ Despite these expected differences between the two methods, the resulting maps can be remarkably similar (e.g., 23). We illustrate this here with maps obtained for the protein groEL. Figure 1a shows a density near residues 10–170 for a crystallographic map of groEL at a resolution of 3.8 Å using experimental (MIR) phase determination followed by density modification. The MIR data used for PDB entry 1oeL^25^ were reanalyzed at a resolution of 3.8 Å to create this map. As an atomic model was not used to obtain this X‐ray map, it can be thought of as an “experimental” map (without bias from an atomic model). Figure 1b shows another map for density near the same residues 10–170 of groEL, this one obtained with cryo‐EM at a resolution of 3.5 Å EMDB, (Electron Microscopy Data Bank, entry 8,750, PDB entry 5w0s).^26^, ^27^ This map is also experimentally determined and does not have any model bias.

In comparing the maps, it should be noted that the resolution dependencies of X‐ray maps are typically determined by the resolution dependence of the experimental data (the maps are often not sharpened or blurred), while those of cryo‐EM maps are determined largely by the resolution dependencies of the accuracy of the data (the maps are typically sharpened or blurred based on agreement between two half‐maps or other measures of map quality as a function of resolution).

The two maps in Figure 1 are not identical, but they have detailed features in common including the locations of many side chains and very similar continuous density corresponding to the main chain (we will refer to the features of these maps as “density” for convenience, but cryo‐EM maps represent electric potential and X‐ray maps represent the density of electrons). On close inspection, some of the differences include missing density at positions of negatively charged side chains (aspartyl and glutamyl residues, see arrows marking three aspartyl residues in Figure 1a,b) in the cryo‐EM map as has been reported.^23^, ^28^ Overall, it might be expected that maps such as these could be interpreted by the same software, perhaps with adjustments for differing appearances of some side chains between the two methods.

The contour levels for the two maps in Figure 1a,b have been adjusted to be the values that just keep the density for the main chain continuous for the entire segments shown in each case. A feature in common for these two maps is that at these contour levels the path of the main chain is almost unambiguous, with no obvious connections through side chains between separate parts of the chain. Although such a clear path of the main chain may not always occur in cryo‐EM or X‐ray maps, main‐chain density is often present at a higher level than side‐chain or noise density. Figure 1c,d illustrates this for these maps by showing the density in the same maps as illustrated in Figure 1a,b, but at higher contour levels where the main‐chain density remains strong in most (but not all) places and side‐chain and noise density are considerably reduced.

Here we make use of the relatively continuous density present for the main chain of proteins to develop a method for automatic interpretation of cryo‐EM maps that combines shape information in cryo‐EM maps obtained at varying contour levels to trace the path of the main chains of proteins and to build atomic models representing them.

## 2 RESULTS AND DISCUSSION

### 2.1 *Tracing protein chains in a cryo‐EM map (*phenix.trace_and_build*)*

Our approach for identifying the path of the main chain of a protein in a cryo‐EM map is to locate helices and strands and then to connect appropriate ends by finding the path that has the highest minimum density in the map. This approach is very similar to the way that an experienced structural biologist would interpret an X‐ray or cryo‐EM map using a visual tool such as *Coot*.^7^ The researcher might first identify secondary structure in the map using a high contour level for visualization. Then gradually lowering the contour level, the researcher would see density representing the main chain of the protein begin to extend from the secondary structure elements. At some point, these tubes of density might just connect with other regions of density. In many cases, the resulting path through the density, which can be thought of as the path with the highest minimum density, will show the main chain of the protein between those secondary structure elements (rather than one through side chains). A similar approach can be used to find extensions to any structure already built and to find segments of a chain without any secondary structure as well.

**Figure 2:**
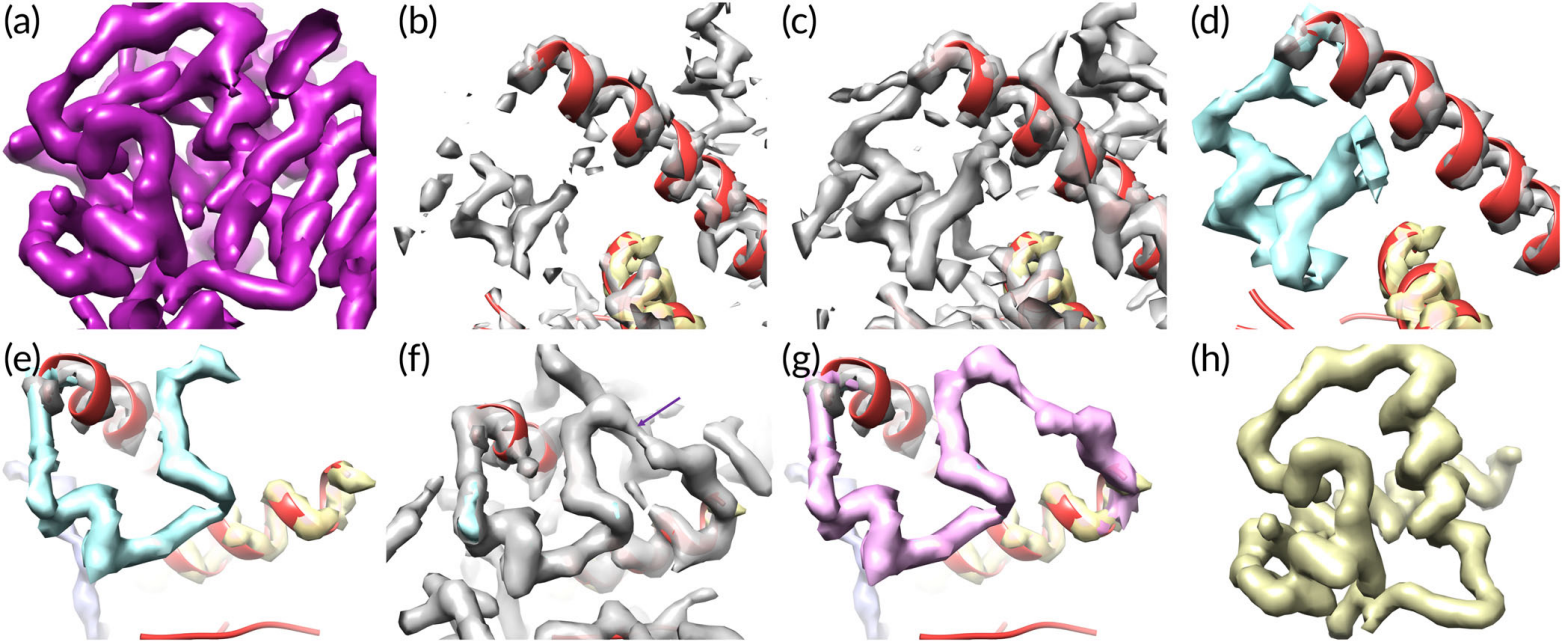
Automatic analysis of density in a map of rotavirus VP6 protein. (a) Box of density cut out from a rotavirus VP6 structure at a resolution of 2.6 Å.^29^ Contours are at an arbitrary level adjusted to maximize continuity of chain (tubes of density) and minimize connections between different parts of the chain. (b) Helical fragments identified in the map shown in (a) (different view) and map density at a high contour level. (c) View as in panel B, but density at a lower contour level showing connections to the helical fragments. (d) Interpretation of density in panel C, where density of one color (e.g., blue) corresponds to points that are connected at a particular contour level and not already part of a previously‐identified region (e.g., the gray density). (e), (f), and (g) Connecting segments of density by gradually decreasing contour level. Panel (e) shows a set of segments (grey, blue, yellow) identified in previous steps. Panel (f) shows density in the map at a slightly lower contour level that just connects the blue and yellow segments. Panel (g) shows the automatic reinterpretation of the blue and connecting density as a single purple segment. (h) Density representing one isolated chain obtained from the map shown in panel (a) using the automatic procedure described here (see text)

Figure 2 illustrates this procedure for chain tracing applied automatically to a small box of density cut out from a cryo‐EM map of a rotavirus VP6 structure reconstructed at a resolution of 2.6 Å.^29^ Figure 2a shows a box of density contoured at a level chosen to show continuous density for protein chains that are present. The box of density contains density corresponding to several non‐contiguous segments of the protein, and the goal is to isolate density for one single continuous chain. In the first step, helices and strands are automatically identified using procedures previously applied to crystallographic maps.^30^ Figure 2b shows parts of two helical fragments that are found in this way. Figure 2b also shows the density in the map at a very high contour level. As is often the case, the density for the helices is higher than the density in most regions of the map, though this is not essential for the procedure.

The key next step is to gradually lower the contour level so that density extends away from already‐identified density. The lowering of contours is stopped before any branching of density occurs, and is also stopped when two regions become connected. The criterion of keeping the contour level high enough that no branching occurs is crucial to the method. Branching typically occurs when the contour level is low enough that side‐chain density for one chain becomes connected to side‐chain density for another chain, yielding two choices for the path of the chain. Keeping the contour level high enough that branching does not occur typically shows the main chains of proteins. In some cases, a true branch occurs (such as disulfide bridges or covalently‐attached ligands). If detected in the current approach, such branches remain as separate fragments. Figure 2c shows that if the contour level is lowered slightly, density extends away from the upper helix, and Figure 2d shows that this can be automatically identified as a single connected region of density (in blue).

Figure 2e–g illustrates two regions of density becoming connected by gradually lowering the contour level. In panel e, segments identified in previous steps are shown. Then in panel f, the density in the map at a lower contour level is displayed and it can be seen that density at this level just connects the blue and yellow segments. Panel g shows the automatic interpretation of this as a new purple segment. Finally, Figure 2h shows the single chain that is identified using these procedures.

Note that although in this example secondary structure was identified as the first step in tracing the chain, the procedure could begin with any starting segments of density. For example, the highest density in the map could be used to identify starting segments.

### 2.2 Reduced representations of the density in the map

**Figure 3:**
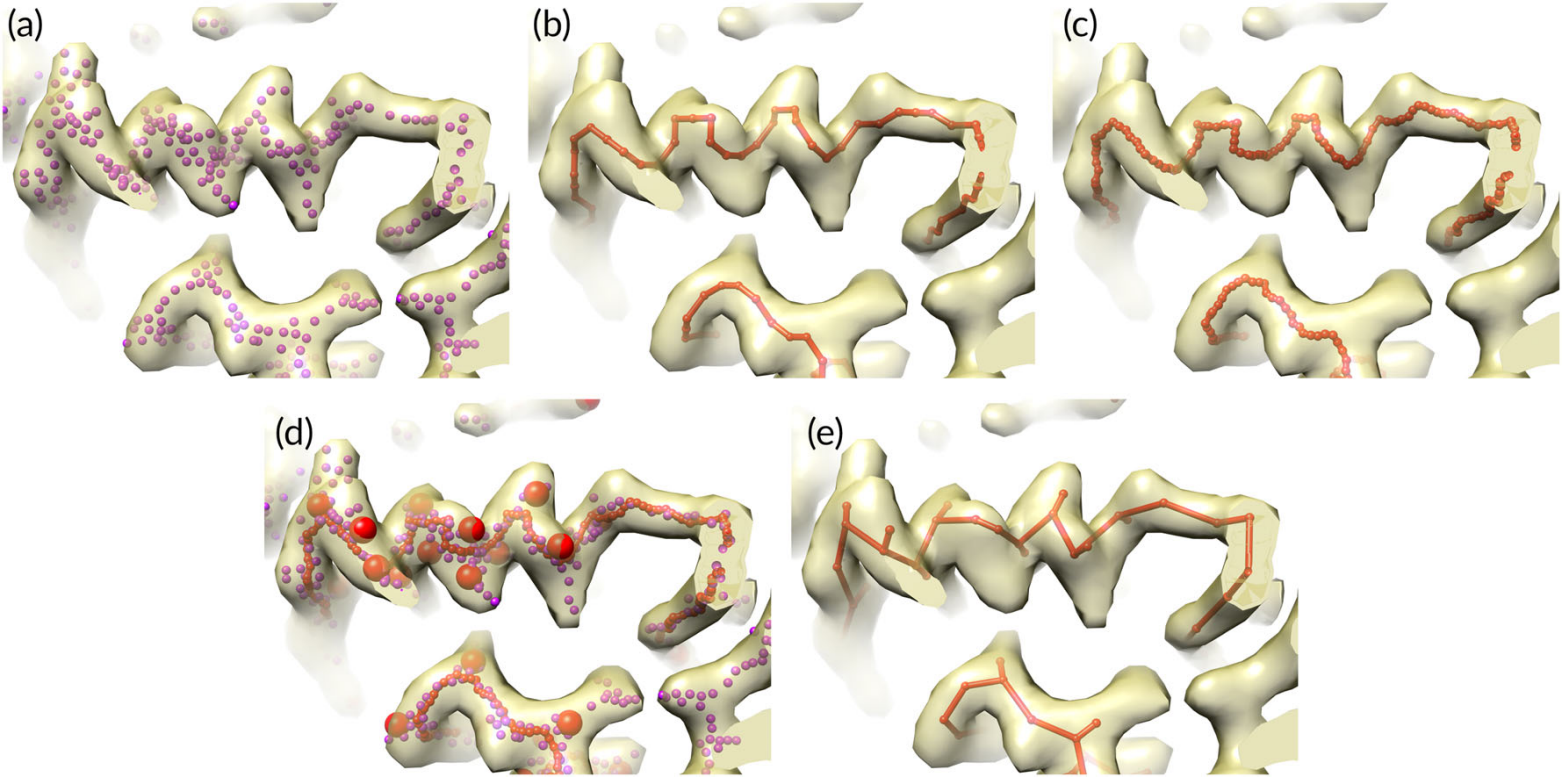
Interpretation of density in rotavirus VP6 map from Figure 2. (a) Bones points (purple dots) marking high density in portion of rotavirus VP6 map. (b) Path points (red points and lines) through high density in map. (c) Path points (red dots) after morphing. (d) Bones points (purple dots), morphed path points (small red dots), and C_β_ positions identified from bones points (large red spheres). (e) C_α_/C_β_ model derived from path points and C_β_ positions

Once a region of density in a map representing a fragment of main‐chain of a protein is identified, two different reduced representations of the density in the map are created to assist in map interpretation. One is related to the “bones” representations that have been used for many years in crystallographic map interpretation,^31^, ^32^ except that instead of the points being on map grid points, they are adjusted to be along the ridgelines of high density (here ridgelines of high density are curves that trace through local maxima in the map in two dimensions nearly perpendicular to the line; they are similar to ridges in a mountain range connecting peaks,^33^ Methods). Figure 3a illustrates these “bones” points (another name for these ridgelines that has been used previously^31^, ^32^) marking high density for part of the rotavirus map shown in Figure 2a. Both the main chain and side chains are visible in this representation. Note that the points in this representation are unordered so that connectivity is not defined by the bones.

The second reduced representation used in generation of a C_α_ model is a simple path through the density representing the fragment of main chain. This path of the main chain through a region of density is constructed based on the weighted minimum‐length path through the density, where the weighting is designed to induce the path to go through high density wherever possible (see Section 3). Such a path for the region shown in Figure 2h is illustrated in Figure 3b.

The path through the density is then adjusted to move it even closer to high density (if possible) using a morphing procedure^34^ designed to move points in the path as close as possible to a nearby “bones” point. This strategy is used instead of moving the points to the highest density because it is difficult to know how far the points should be moved to place them in high density and the bones points have already been moved to ridgelines of density so they are relatively robust markers of where the path should go. In the morphing procedure, the shift for each point along the path is the average of shifts that would move that point and neighboring points to a nearby bones point. Typically, for a particular point, that point and 5 points on either side are considered. For each of these 11 points, the nearest bones point is identified and the shift that would be required to move it there is noted. The average of all these shifts is then applied to the point of interest. This procedure is then repeated for all other points along the path. Figure 3c shows the path in Figure 3b after morphing.

The two reduced representations of the map contain complementary information that can be used in generation of a C_α_ model. The bones points in Figure 3a show both main and side chains, but (in our implementation) the points are unordered and connectivity of the chain is not defined. The path tracing in Figure 3c shows the connectivity of the main‐chain, but does not show side chains.

### 2.3 Construction of a C_α/_ C_β_ model based on one continuous region of density

Once density for a fragment of chain is identified and the bones and path representations of the density are generated, a C_α_ model for that fragment is constructed in three steps. In the first step, likely C_β_ positions are identified based on the presence of bones points extending away from the path tracing (Section 3, Figure 3d). In the second step, C_α_ positions are chosen so as to optimize proximity to C_β_ positions and to optimize distances between C_α_ positions (Section 3). In the third step, any parts of the C_α_ model that overlap with a helix or strand identified in the initial search for secondary structure are replaced with that helix or strand. The final C_α_/C_β_ model for this fragment of a chain is shown in Figure 3e.

### 2.4 Model completion and refinement

Once a C_α/_ C_β_ model has been constructed, we use the reconstruction tool *Pulchra* 35 to create all‐atom models in both directions, we add whichever side chains fit the density best with the *Phenix* 36 tool *phenix.replace_side_chains*,^37^, ^38^ and we refine the resulting model with the *phenix.real_space_refine* algorithm.^39^ The chains in the two directions are scored based on H‐bonding and fit of the model (including side chains) to density, and the higher‐scoring direction is kept (see Section 3). Following this, all the chains in the model are assembled using the *Phenix* tool *phenix.combine_models* 38 which ranks chains based on their length and fit to the density and removes overlapping parts of lower‐ranked chains.

**Figure 4:**
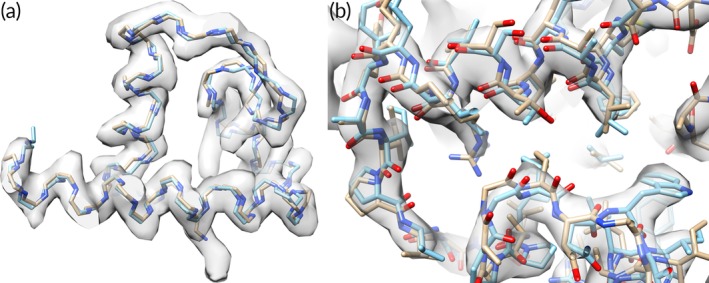
Comparison of deposited and automatically‐built rotavirus models. (a) Comparison of backbone tracing of deposited rotavirus structure (blue) with automatically‐generated tracing (yellow). (b) Detail of comparison of deposited rotavirus structure (blue, PDB entry 3j9s, 29) with automatically‐determined model (yellow)

Up to this point, side chains have been built based on the density in the map. Once a relatively complete model has been created, the sequence is aligned to the model using the tool *phenix.sequence_from_map*.^38^ This tool attempts to identify the amino acid at each position in the model based on the side‐chain density, carries out a sequence alignment to the known sequence, and creates a new model using the aligned sequence. Figure 4a compares the backbone of the resulting model (yellow) with the corresponding part of the backbone of the deposited structure (blue). It can be seen that much of the backbone is similar in the two models, but there are some places where there may be insertions or deletions in the automatically‐generated model. Figure 4b illustrates a small portion of the map and compares the deposited and automatically‐built models. The models are quite similar overall (the C_α_ rmsd between the models is 1.0 Å) but some side chains are in different positions.

### 2.5 Integration into the *phenix.map_to_model* procedure

The *phenix.trace_and_build* method described so far is designed to interpret a map that contains one or more protein chains, but it does not have the capability for analyzing symmetry, sharpening, different chain types (RNA, DNA, protein), or the capability of aligning chains to several sequences in a sequence file. These additional capabilities are made available by incorporating the *phenix.trace_and_build* method into the tool *phenix.map_to_model* which already has them.^20^ For example, the *phenix.map_to_model* procedure can build RNA and protein into a map and choose which chain type best fits each part of the density.^20^ After completion of the *phenix.trace_and_build* procedure, the *phenix.map_to_model* tool carries out three final model completion steps. First, the tool *phenix.fit_loops* 36 is used to fill in gaps, then the tool *phenix.assign_sequence* 36 is used to optimize the sequence alignment, and last, a final refinement is carried out.

### 2.6 Phenix.map_to_model *performance comparing previous model‐building with* phenix.trace_and_build

**Figure 5:**
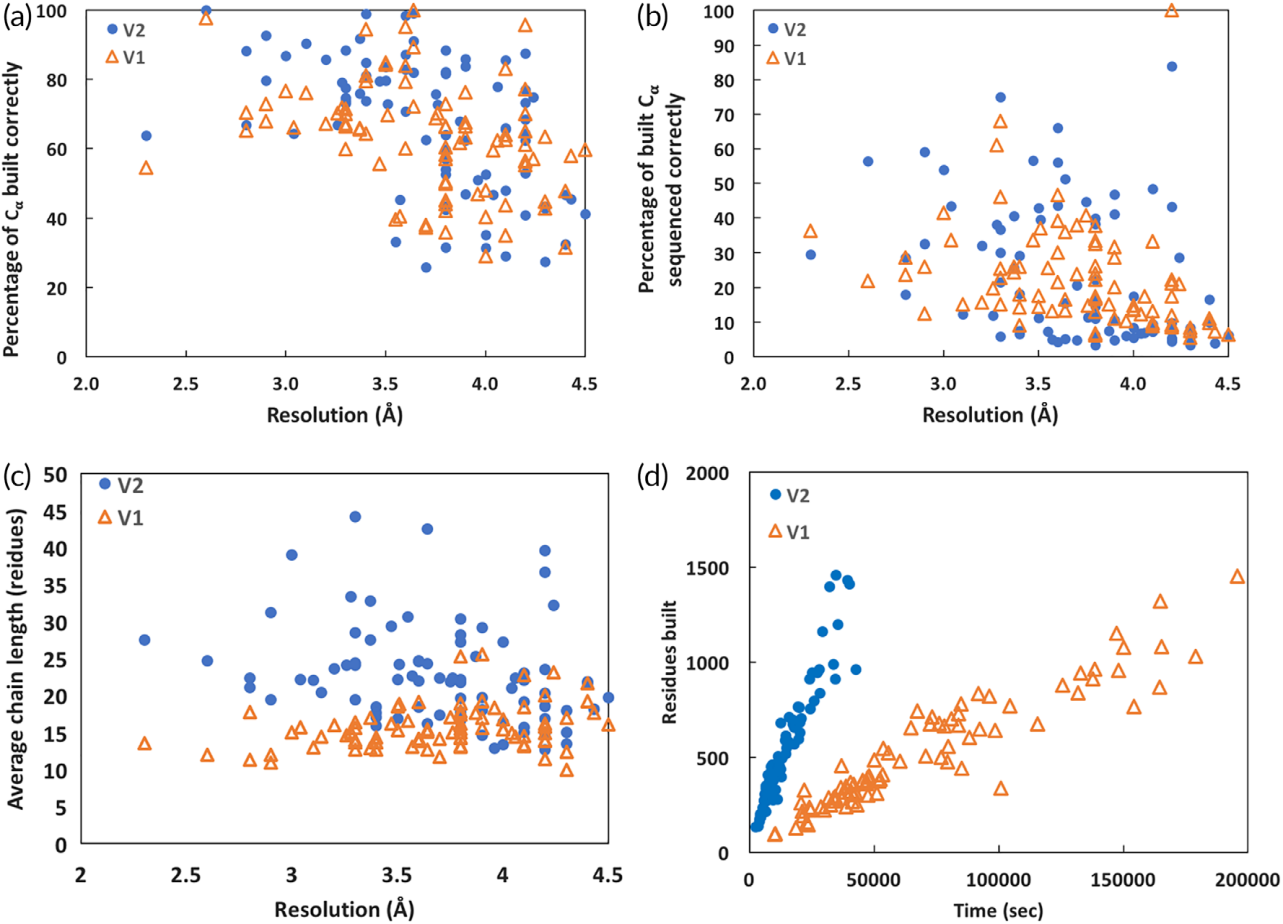
Application of *phenix.map_to_model* procedure to deposited maps and comparison with deposited models. (a) Percentage of C_α_ atoms in each of 88 deposited structures matched within 3 Å by a C_α_ atom in an automatically‐built structure (see text for details). (b) Percentage of side chains for each of 88 automatically‐built models matching the amino acid in the corresponding deposited structure (see text for details). (c) Mean chain length (length of segments between breaks in the chain) for each of 88 automatically‐built models. (d) Time required and number of residues built for automatic model‐building. Triangles are for *phenix.map_to_model* version 1 and filled circles are version 2

The *phenix.map_to_model* tool can use either the previously‐available *Phenix* model‐building methods^20^ which are largely based on RESOLVE model‐building,^40^ or the *phenix.trace_and_build* procedure described here. We compared the performance of the two procedures by applying each method to 88 maps from the EMDB with resolutions from 2 Å to 4.5 Å, and using the corresponding deposited models for comparison. In each case the *phenix.map_box* tool was used with the “extract_unique” keyword and the symmetry of the map to isolate the density for the asymmetric unit of the map as described,^38^ and the *phenix.map_to_model* procedure was applied using either the previous RESOLVE model‐building approaches (version 1) or the *phenix.trace_and_build* procedure (version 2). The overall success rate of model‐building using the two approaches is similar. Figure 5a shows the percentage of C_α_ atoms in the deposited structure that are reproduced (within 3 Å, as determined using the *Phenix* tool *chain_comparison*) using each method. The average fraction matched with version 1 is 63%, while for version 2 it is slightly higher (66%). For both methods the fraction built decreases considerably for maps with resolution worse than about 3.5 Å. Figure 5b illustrates the fraction of the residues built in each case that are assigned to the correct amino acid type as reported in the deposited model. Both methods again have better success at resolutions better than 3.5 Å than at lower resolutions. The mean percentage sequenced correctly is 23% in both cases.

The results of applying the two methods differ, however, in two important respects. Figure 5c shows that the mean length of chains built using *phenix.map_to_model* version 2 is greater than those built with version 1, with a mean chain length of 23 for version 2 and 16 for version 1. The longer chain length means that the resulting model has fewer chain breaks and will require fewer choices during sequence alignment. Figure 5d shows that the time required to produce a model is substantially less for version 2 than version 1. In both cases, the time required for model‐building is very closely proportional to the number of residues built. The mean time per residue built for version 1 is 131 seconds, while for version 2 it is 27 seconds. This four‐fold improvement in speed means not only that a researcher can obtain a model more quickly with version 2 of *phenix.map_to_model* but also that it becomes feasible to try more possibilities during model‐building in an effort to increase the fraction of the model that is built. Supporting Information Spreadsheet I contains additional information about the models built. In particular, it lists the number of residues built in the forward direction, reverse direction, and mixed which reflects the ability of the method to distinguish chain direction. These vary considerably. For example, the analysis of EMD 8191 at 2.8 resulted in ¾ of the residues built in the forward direction while EMD 8574 at the same resolution resulted in only 1/3 built in the forward direction.

The methods used in version 1 of *phenix.map_to_model* are all available as well in version 2. Version 2 can be run in a “quick” mode in which only the new methods described here are run. This is typically takes about half the time as a standard run. Additionally version 2 can be run in a “thorough” mode in which all the methods from version 1 are run as well as the new approaches, the resulting models are all scored (see Section 3) and the highest‐scoring model is kept. All these options can be chosen by the user and various approaches can be tested for challenging structures.

**Figure 6:**
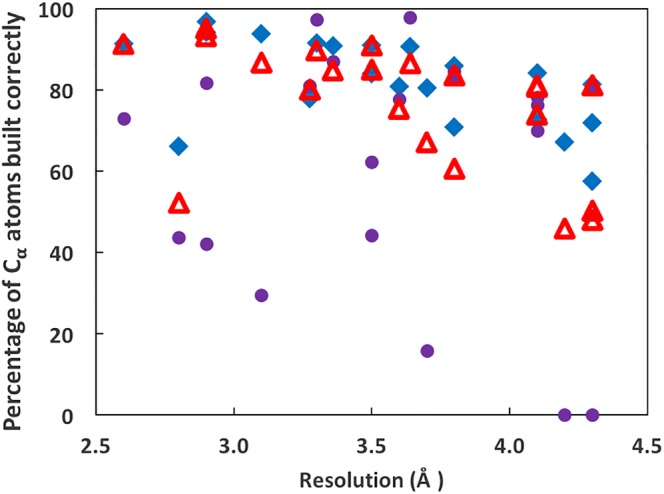
Comparison of *phenix.map_to_model*, *MAINMAST*, and *Rosetta* model‐building procedures. Percentage of C_α_ atoms in each of 22 deposited structures matched within 3 Å by a C_α_ atom in an automatically‐built structure (see text for details). Solid diamonds are for *MAINMAST*, closed circles are *Rosetta*, and open triangles are *phenix.map_to_model* version 2

We also compared the *phenix.trace_and_build* model‐building procedure (as integrated into *phenix.map_to_model)* with two other model‐building procedures, *Rosetta* model‐building^14^ and *MAINMAST* model‐building.^41^ We recently compared the *Rosetta* and *MAINMAST* procedures with version 1 of map_to_model^20^ using 22 maps at resolutions ranging from 2.6 to 4.3 Å. Here, we use the same maps and *Rosetta* and *MAINMAST* models^41^ as we used in that analysis and compare them with models obtained using version 2 of *phenix.map_to_model*. Figure 6 shows the percentage of C_α_ atoms in each deposited structure that are reproduced within 3 Å (as in Figure 5a) by each method. Overall, the *phenix.map_to_model* procedure reproduced 77% of the residues in these models, *MAINMAST* reproduced slightly more (82%), and Rosetta reproduced somewhat less (63%). The rms positional difference between C_α_ atoms in the deposited structures and *phenix.map_to_model* models was 1.35 Å, while for *MAINMAST* models it was somewhat higher (1.51 Å) and for *Rosetta* models it was slightly lower (1.31 Å).

**Figure 7:**
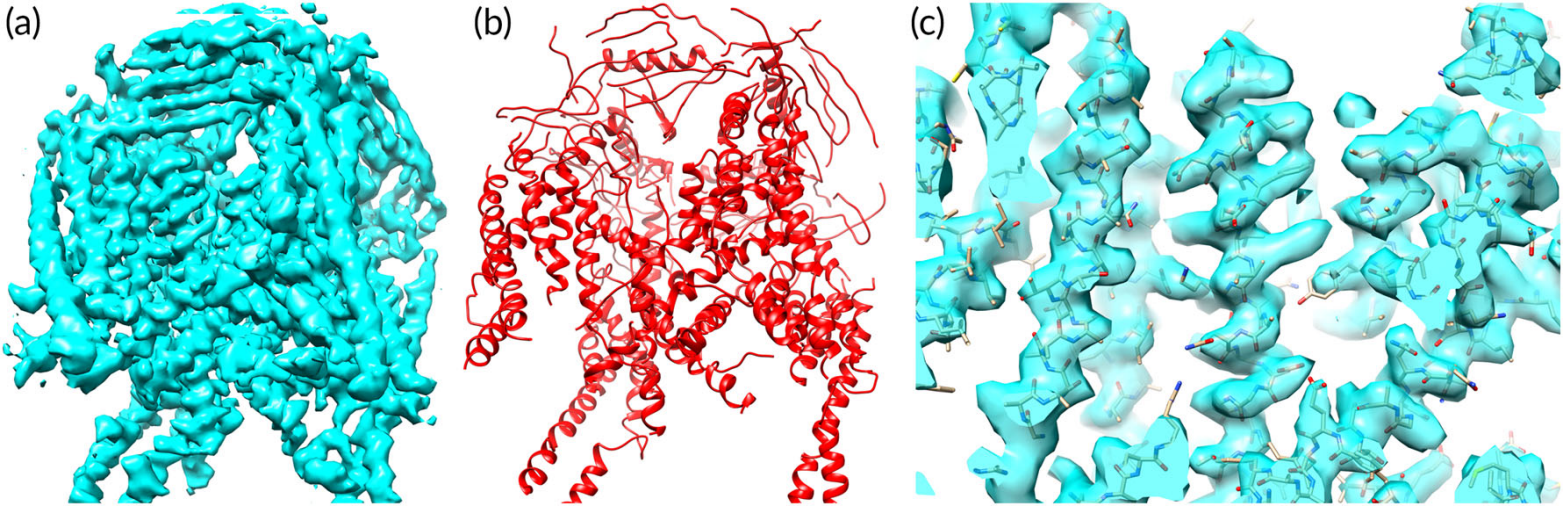
Models created by *phenix.trace_and_build* model‐building procedure. Results of applying the *phenix.trace_and_build* model‐building procedure to the human endolysosomal TRPML3 channel (EMD 7018, PDB entry 6aye) at a resolution of 4.1 Å.^33^ Panel (a) shows the sharpened map, (b) illustrates the model that is automatically built using default parameters in *phenix.map_to_model*, and (c) shows a detail of the model and map

**Figure 8:**
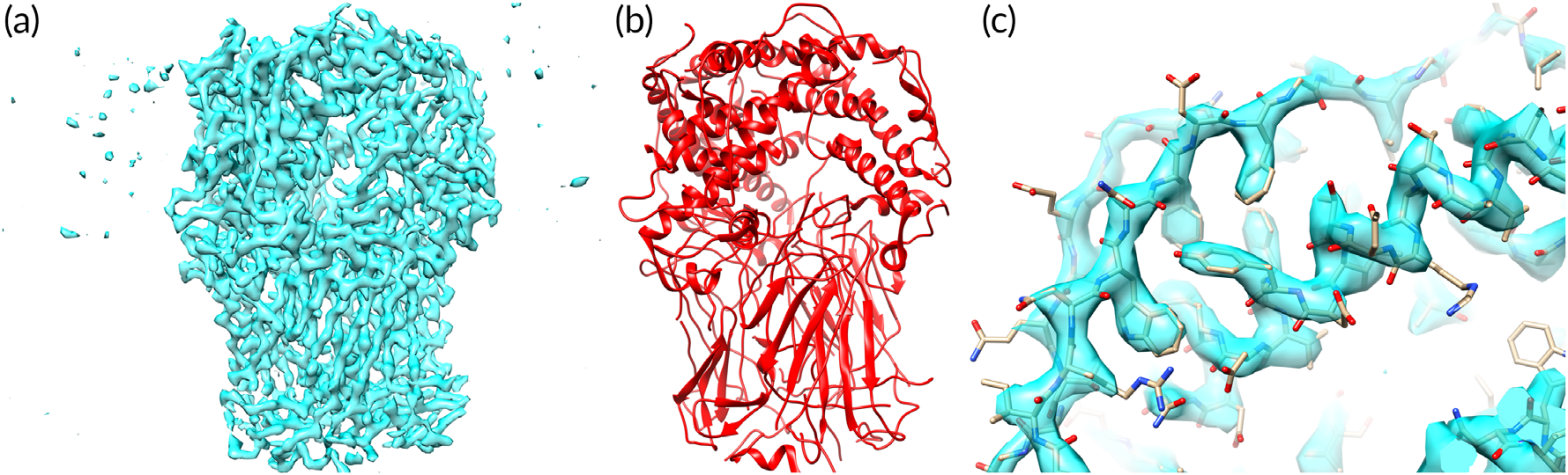
Model‐building at a higher resolution. Panel (a) shows a sharpened map for rotavirus VP6 (EMD 6272, PDB entry 3j9s) at a resolution of 2.6 Å.^29^ Panel (b) shows the model that is built using *phenix.map_to_model*. Panel (c) shows a detail of the model and map

Figures 7 and 8 illustrate two specific examples of model‐building with *phenix.map_to_model*. In Figure 7 the *phenix.trace_and_build* model‐building procedure is applied to the human endolysosomal TRPML3 channel (EMD 7018, PDB entry 6aye) at a resolution of 4.1 Å.^42^ At this resolution many details are not visible in the map and there are some places where mis‐tracing is visible. Figure 8 shows a sharpened map and model for rotavirus VP6 (EMD 6272, PDB entry 3j9s) at a higher resolution of 2.6 Å.^29^ At this resolution the map shows considerably more detail than in the lower‐resolution map in Figure 7 and the model is considerably more accurate.

### 2.7 Conclusions

We find that our *phenix.map_to_model* procedure for model‐building, closely following the way an experienced structural biologist would approach the problem, is an effective and rapid model‐building procedure. The procedure is comparable to existing methods in the completeness and accuracy of models that are produced, it is four times faster than our previous *phenix.map_to_model* procedure, and it produces models with fewer chain breaks than our previous procedure. The main reason why the new procedure is faster than our previous methods is the use of the connectivity tool to trace density at a particular contour level. This approach is much quicker than the extensive testing of fragments as possible extensions of a helices and strands that were used in our previous methods. Additionally, the new procedure has many steps that can be run in parallel, so the use of multiprocessing substantially shortens the clock time required to run. We expect that this method will be generally useful for structural biologists in obtaining preliminary ab initio models from cryo‐EM maps at resolutions in the range of 2 to 4.5 Å. It should be emphasized that the models obtained using the methods described here are not producing final models ready for deposition. For maps at resolutions of about 3 Å and better, the models from these and other approaches are relatively complete, but even these still need substantial manual work to complete. Normally a researcher would use automated tools such as those described here to get a preliminary model, then graphical tools such as *Coot* would be used to build additional model by hand. These models would normally then be refined and used as input for additional rounds of manual or automated model‐building before deposition.

Finally, an important aspect of the current method is that it is largely orthogonal to previous methods in that it uses a direct tracing of the density as the main approach for creating a polypeptide chain. This means that it can be used in combination with other methods to create an ensemble of models that collectively represents a range of plausible interpretations of a map.^20^ This can be used to estimate a lower bound on uncertainties in a model^43^, ^44^ and to create density corresponding to an overall best estimate from all models.

## 3 METHODS

### 3.1 Maps and models used

The 88 cryo‐EM maps used in Figure 5 were chosen to represent a range of resolutions from 2 Å to 4.5 Å. A list of the maps and models and a summary of the results obtained for each is in Supporting Information Spreadsheet I. The maps were obtained from the EMDB and were automatically sharpened using the *phenix.auto_sharpen* procedure^45^ which requires only a map and an estimate of resolution (taken from the EMDB and PDB meta‐data). The symmetry in each map was automatically identified with the *phenix.map_symmetry* tool,^38^ requiring again only a map and a resolution estimate. This symmetry was used with the *phenix.map_box *tool to extract the unique part of the map,^38^ creating a small sharpened map just containing this unique part that was used for all further work.

The model corresponding to each map was obtained from the PDB and if necessary, the symmetry obtained above was applied to generate a model that covered at least the unique part of the map.

The 22 maps and corresponding *Rosetta* and *MAINMAST* models in Figure 6 were the same maps and models used in the analysis described previously.^20^

### 3.2 Connectivity analysis

We use the *Phenix* connectivity tool^45^ to identify regions consisting of all grid points in a map that are all above a threshold (the contour level) and that all are adjacent to another grid point in the same region. For a particular contour level, the connectivity tool produces a list of regions, and for each region, it produces a list of all points in that region. The tool is particularly useful in the present work because it can be used to rapidly determine whether two points (e.g., one at the end of one helix and one at the beginning of another) are in the same region, and therefore are connected at that contour level.

At the beginning of the connectivity analysis, density for the entire map is considered. As regions of density are identified, those regions of density are removed from the map (masked) so that they will no longer be considered in subsequent steps. The very ends of these identified regions of density are not removed so that connections can be made with subsequently‐identified regions. The process starts with the highest density in the map and works down to lower density. At intermediate stages of analysis (after the highest density is identified), much of the high density in the map will be masked out (all the regions that have been identified so far, such as the grey region in Figure 2d), and connections that are weak may become visible at low contour levels.

In our procedure, any regions found that have branching are ignored. This means that if a particular region becomes branched as the contour level is decreased, the region will only be analyzed for higher contour levels where it is not branched. This step is important because branching is usually caused by side‐chain density merging between adjacent chains, so tracing through a branch would cause errors in the main chain interpretation. A key parameter in the connectivity analysis is the choice of length allowed for a branch extending from a chain. In density maps of proteins, side chains extend from the main chain at regular intervals, and their sizes are well‐defined and their lengths are quite short relative to the length of the main‐chain. We use a cutoff of 10 Å as the maximum branching length, chosen empirically to limit both mis‐tracing through side‐chains and fragmentation of the main chain (this and essentially all other parameters are user‐adjustable).

### 3.3 Path tracing

An important step in our procedure is tracing a path from one end of a region of density to the other, following high density as much as possible. We use a variant of Djikstra's algorithm^46^ in which the path between two grid points is weighted based on the density at the second grid point. The weight *w* for a point with density value of *ρ* we used was,

(1) $$w={\left[\left(\;\rho_{\max}–\rho \right)/\left(\rho_{\max}–\rho_{\min}\right)\;\right]}^{a}$$

where *ρ*_min_ and *ρ*_max_ are the minimum and maximum density of any points in the region considered, and the exponent *a* is a parameter with default value of 2. This function has the property that connections between grid points that have values of density close to the maximum value in the region (where we would like to trace the chain) have almost zero weight while those with values near the lowest in the region (which we would like to avoid) have weight near unity. Then tracing the minimum weighted path tends to produce a path from one end to the other using the highest‐density available points along the way.

Using this approach, the path through a region of density is obtained by starting at an arbitrary point A inside the density, finding the furthest point B, then finding the point C furthest from B. Then the (unweighted) length of path between points B and C is the length of the region. The length of a branch is the (unweighted) length of the longest weighted path between a point on that branch and a point on the path between B and C.

### 3.4 Marking ridgelines of density in a map with marker atoms

We have developed a method for protein chain‐tracing^33^ that involved constructing a set of marker atoms that essentially follow the ridgelines (high points) of density in a map. These marker atoms are similar to the “bones” used for many years as indicators of the main‐chain with side‐chains coming off at regular intervals,^31^ except that the atoms are explicitly placed at the highest‐density points along ridgelines, not on grid points. Additionally, the points in our “bones” representation are unordered and do not show the connectivity in the map. In this work, we use these bones atoms for two purposes. One is in morphing the path of the chain to move it into high density, and the other is in identification of locations of side chains.

### 3.5 Identification of likely C_β_ positions

We use a comparison of the “bones” and “path” reduced representations of the map to identify likely C_β_ positions. The “bones” representation (Figure 3a) is first modified to remove all points that are close to a point in the path representation (by default, within 1.5 Å). In this fashion, the modified bones essentially represent the positions of the side chains. Then each point along the path (Figure 3b) is used as a seed, and the centroid of all nearby (by default, within 3 Å) modified bones points is calculated. The resulting set of centroids is clustered and the centers of the clusters are considered as likely C_β_ positions (shown in Figure 3d).

### 3.6 Construction of C_α_/C_β_ model from morphed path points and likely C_β_ positions

A C_α_/C_β_ model is constructed in two steps beginning with the morphed path points and the likely C_β_ positions. In the first step, a starting set of C_α_ positions is generated, and in the second step the positions of these C_α_ atoms are refined.

The starting set of C_α_ positions is chosen from points along the morphed path. The number of C_α_ positions is initially set based on the total length of the morphed path divided by the expected C_α_–C_α_ distance of 3.8 Å. Positions are chosen to be adjacent to the likely C_β_ positions where possible and are spaced approximately 3.8 Å apart along the path. Then C_α_ positions are iteratively inserted between existing sites, deleted, or placed on the ends of the segment, the resulting set of C_α_ positions is scored, and the highest scoring arrangement is kept. The scoring consists of four terms, each of which has a weight that is user‐adjustable and which was set up with minimal experimentation so further optimization of the method is possible. The first term reflects the deviation between C_α_–C_α_ distances and the target of 3.8 Å. The second reflects proximity to an identified C_β_ position. The third reflects the density at mid‐points between C_α_ positions (these positions are expected to be in high density as well). The fourth reflects how close the mid‐points between C_α_ positions are to a point along the path. Each of the scoring terms consists of a weighted sum of squares of deviations between observed and target values. These scoring terms are described below.

Once a set of C_α_ positions is obtained, the coordinates of these sites are refined so as to minimize a function that includes geometrical and density terms, each with a weight that is set by default and that has not been fully optimized. The target function includes a term for deviation from the starting position, a term for deviation from the target C_α_–C_α_ distance of 3.8 Å, and a term for density at C_α_ and C_β_ positions and for density at the mid‐point between C_α_ positions.

### 3.7 Replacement of parts of a C_α_ model with coordinates from a helix or strand identified in secondary structure search

We noticed that in many cases the coordinates of C_α_ positions in the helices and strands identified in a secondary‐structure search of the map are more accurate than those obtained with the methods described so far. Accordingly, an important part of our approach is to splice these helices and strands into the C_α_ model. This is done by identifying positions along the helices/strands and C_α_ model where the two sets of C_α_ atoms nearly overlap (typically within ¾ of the target C_α_–C_α_ distance) and allowing a crossover at that position.

### 3.8 Scoring models based on hydrogen bonding, overall fit to density, fit of side chains to density, and uniqueness of side chain fitting

We use five characteristics of a model in scoring. The first is the presence of H‐bonding along the main‐chain of the model. The tool *phenix.find_ss_from_ca* is used to find secondary structure elements in the model and to identify likely hydrogen bonds in the model. The score is the number of good hydrogen bonds (heavy atoms within 3.5 Å) plus half the number of poor ones (where the two hydrogen‐bonding partners are part of a helical or beta‐sheet arrangement but further than 3.5 Å apart, with a default weight of unity.

The second scoring term is based on fit of the model to the density, and is given by,

(2) $$U=10\;\left(\mathrm{CC}-0.5\right)\;N^{½}$$

Where *U* is the score with a default weight of unity, CC is the value of the correlation between map density and model‐based density in the region of the model, and *N* is the number of atoms in the model. The scale of 10 is arbitrary and set so that the default weight can be unity. This scoring function has the property that a model with more atoms has a higher score than one with fewer atoms, if all else is the same. This favors models that are both more complete and a better fit to density.

The third through fifth terms in scoring are based on the fit of side chains to density. The *Phenix* tool *phenix.replace_side_chains* is used to identify the side chain and rotamer that best fits the map at each amino acid position. This procedure is the same as used previously in crystallographic model‐building.^37^ In the process, a relative probability, a log‐likelihood score based on that probability, and a *Z*‐score based on the distribution of log‐likelihood scores are calculated for each amino acid at each position. The sum of the log‐likelihood scores for the final fitted amino acids is the third score for that model with a default weight of unity.

The fourth term is based on the uniqueness of the fit of amino side chains to the density. If the most likely amino acid at a particular position is less than (by default) 1% more likely than the least likely, or if the *Z*‐score for the most likely amino acid is less than (by default) 0.5, the position is considered to not have a unique identification of a side chain and the (penalty) score is the number of such non‐unique positions, with a default weight of –¼.

The fifth term is another measure of uniqueness. It is the number of positions where glycine is the most likely, minus the approximate number expected based simply on amino acid composition (about 7.4% by default), with a default weight of –½.

As in the previous scoring schemes, each of these five terms is weighted by an empirically‐set weight that is user‐adjustable and has not been extensively optimized.

### 3.9 Side chain identification based on density at expected side‐chain positions

We used the approaches developed earlier for interpretation of side chain density in X‐ray maps to obtain probabilistic estimates of the side chain present at each position along a model built into a cryo‐EM map.^37^ In essence, the procedure consists of using the coordinate of the main‐chain atoms at a particular position in the chain to predict the location of the side chain, considering the conformations of each member of a rotamer library^47^ representing likely side chain conformations. For each rotamer of each side chain, the density in the map is compared (by correlation coefficient) with average density obtained in a test set of maps for that rotamer and side chain. One conformation is tested for each rotamer. For each amino acid type, the rotamer with the highest correlation between expected and observed density is chosen. Then the various amino acid types are weighted based on their density correlations.^37^ If no sequence information is available, the most likely amino acid is chosen for each position and the best‐fitting rotamer for that side chain is built. If sequence information is available, the sequence that has the overall highest likelihood based on the density correlations for all the residues in a chain is used to identify the sequence of that chain.^37^ At each position in the chain the amino acid is chosen based on the fitted sequence and the best‐fitting rotamer for that amino acid at that position is built.

For this work, we used the side‐chain rotamer library previously generated using X‐ray structures,^37^ and updated the expected density for each rotamer of each side chain using 165 deposited cryo‐EM maps and models with resolutions from 2.2–4.0 Å (22 of these 165 maps used to generate side chain density are among the 88 structures analyzed in Figure 5).

### 3.10 Options for quick, medium, or thorough model‐building

In our development of the *phenix.trace_and_build* procedure, many choices were made for default values of parameters and perhaps more importantly for whether a specific step is included in the procedure. These choices were typically based on tests with one or a few maps, and occasionally were based on tests with all 88 maps shown in Figure 5 (though not using the 22 maps in Figure 6). The default choices were also based on the amount of computation required, with default values often chosen to be those that required the least computation. In order to give a user flexibility in the amount of computation to invest in model‐building, we included in *phenix.map_to_model* four overall options for thoroughness of model‐building. In the *quick* version, *phenix.trace_and_build* parameters are set to minimize the total computation required. In the default *medium* version, *phenix.trace_and_build* is run with parameters set to maximize the amount of model built. In the *thorough* version, *phenix.trace_and_build* is run once with the *quick* version of parameters, and then a second time with the *medium* version, and the resulting models are combined and the parts that fit the map from each are extracted with the *phenix.combine_models* algorithm.^20^ In the *extra_thorough* version, the quick and medium versions are each run twice, using the result from the first run to optimize the map sharpening^45^ and including the result from the first run in the model‐building process for the second run, producing four models. From these four models the one that has the highest combined score is chosen. The combined score (*T*) is the sum of the likelihood score *S* from the fit of sequence to the density^38^ and a map correlation score given by the map‐model correlation *CC* multiplied by the square root of the number of residues built (*N*):

(3) $$T=S+\mathrm{CC}\;N^{½}$$

In this equation the likelihood score for fit of sequence to density (*S*)^38^ is based on the match of each side chain to density using the chosen sequence relative to the side chain match to density for all other possible side chains.

## Supporting information


**Appendix S1**: Supplementary Information
